# Prepare to fail or failing to prepare? Acute performance after the 11+ with and without strength exercises

**DOI:** 10.1136/bmjsem-2023-001634

**Published:** 2023-11-02

**Authors:** Varg Ringdal Støvland, Roar Amundsen, Gøran Paulsen, Torstein Dalen-Lorentsen

**Affiliations:** 1 Department of Sports Medicine, Norwegian School of Sports Sciences, Oslo, Norway; 2 Department of Physical Performance, Norwegian School of Sports Sciences, Oslo, Norway; 3 Department of Smart Sensors and Microsystems, SINTEF Digital, Trondheim, Norway

**Keywords:** Soccer, preparation, resistance exercise, health, planning, periodisation, FIFA 11+, 11+, warm up, session

## Abstract

**Objectives:**

The 11+ is an effective injury prevention warm-up programme but is often poorly adopted in practice. One reason for low compliance is the claim that the strength training part of the programme acutely impairs muscle performance before the football activity. This study aims to compare the acute effects of the 11+ with (WU+S) or without (WU-S) the strength training part on performance.

**Methods:**

Fifteen female junior football players completed WU+S and WU-S on two separate days in randomised order. Maximal voluntary torque in knee extension and flexion (60°/s and 180°/s) and countermovement jump (CMJ) were tested before and after performing the warm-up protocol. Sprint performance and rating of perceived exertion (RPE) were assessed post-warm-up.

**Results:**

Warm-up with strength training reduced peak torque in knee flexion at 180°/s more than WU-S, while no differences were found at 60°/s. Knee extension work was reduced more with WU+S than WU-S at 180°/s, but no differences at 60°/s. Peak torque angle and CMJ were unaffected. Players were slower on 20 and 30 m sprints after WU+S than WU-S. The RPE was higher after WU+S than WU-S, but there were no differences in readiness to train between the two protocols.

**Conclusion:**

Performing the 11+ programme as a warm-up routine with the strength training part can impair subsequent knee flexion torque at high velocity and sprint performance in female junior football players compared with performing the 11+ warm-up without the strength part.

**WHAT IS ALREADY KNOWN ON THIS TOPIC**

- The 11+ warm-up programme can reduce the risk of injuries, but compliance with the programme is low.
- Time constraints and a fear of impairing subsequent performance are perceived barriers to compliance with the programme. The strength training part of the programme composes most of the time needed to perform the programme and likely causes an acute decline in muscle function.
- Performing the strength training part of the programme after training is associated with higher compliance with the full programme and similar preventative effects.

**WHAT THIS STUDY ADDS**

- Performing the 11+ warm-up with the strength training part can impair subsequent physical performance. Players sprinted faster and reported lower ratings of perceived exertion acutely after performing the 11+ without the strength training part compared with the strength training part.

**HOW THIS STUDY MIGHT AFFECT RESEARCH, PRACTICE OR POLICY**

- The strength training part of the 11+ warm-up programme should be performed after football training or in separate sessions.

## Introduction

The warm-up is a natural start to every training session and is generally accepted practice to enhance athletic performance.^1 2^ Proposed mechanisms for the enhanced performance include increased muscle and tendon flexibility, muscle temperature, blood flow to the extremities and increased contractile function.^3^ On the one hand, a warm-up procedure must be sufficient to elicit positive effects but not cause fatigue and decrease performance on the other.^4^

The warm-up has been targeted for injury preventive measures in the past decades, and several interventions have been developed and tested. In 2006, Fédération Internationale de Football Association’s Medical and Research Centre developed the 11+ injury prevention programme, a warm-up routine that consists of three parts: (1) running exercises, (2) strength, plyometrics and balance and (3) running exercises. Previous studies have shown that the programme can reduce injury risk in football players.^5–7^ The injury preventive effect of the 11+ programme is likely to be twofold: (1) an acute increase in physical and mental preparedness^8^ and (2) a long-term effect of the strength and proprioceptive training programme, that is, players becoming stronger over time.^9 10^

The five strength exercises are an integral part of the 11+. If one expects long-term strength adaptation, the strength training volume must be large enough to elicit adaptation. This can cause a dilemma, as the required strength stimulus needed for adaption may result in an acute decline in muscle function^11^ and thereby be counterproductive for the warm-up—to prepare for the current session. One of the exercises in the programme is the Nordic hamstring exercise, which can reduce the risk of hamstring injuries among football players when performed before and after practice.^12–14^ Given the eccentric high-force nature of the exercise, an acute reduction in maximal hamstring force is likely a consequence.^15 16^ There is, therefore, a need to investigate whether the strength training part in the 11+ affects acute performance negatively.

The acute effects of the complete 11+ programme have previously been explored^17–20^ but never in a population of female adolescent players. The risk of injury can differ based on sex, playing level and age,^21^ and there is currently a lack of research on female football players.

Therefore, we aimed to investigate if the full 11+ programme negatively affects physical performance compared with a shorter version of the programme without the strength training part. We hypothesise that there would be a significant difference in the acute effects between the two protocols in favour of the shorter version based on the type of exercises and duration in the strength training part.

## Methods

We invited 20 female youth football players (15.7±1.6 years.) to participate in this randomised cross-over study by contacting the head of youth development and head coach of two teams at the elite and subelite levels. Female youth football players were chosen as they are a neglected population (in research), although they are a target population for the 11+ programme. Players performed the 11+ warm-up protocol (table 1) with (WU+S) or without the strength training part (WU-S) on separate days (2–4 days between tests) in randomised order. Strength and jump height were assessed before and after the warm-up protocols, while sprint performance, rating of perceived exertion (RPE) and readiness to train were assessed after the warm-up protocols. Players (and parents of players <16 years) gave informed consent to participate. They could not have had a time-loss injury the 2 weeks before the project or have trained in the Nordic hamstring exercise regularly through the season, assessed verbally during inclusion.

**Table 1:** The 11+ warm-up protocol

11+	
Duration	
Part 1: Running exercises	8 min
Straight ahead	2 sets over 30 m each exercise
Hip out	
Hip in	
Circling partner	
Shoulder contact	
Quick forwards and backwards	
Part 2: Strength	10 min
The bench: static	3 sets×20–30 s
Sideways bench: static	3 sets×20–30 s (each side)
Nordic hamstring: beginner	1 set×5 repetitions
Single leg stance, hold the ball	2 sets 30 s (each leg)
Squats with toe raises	2 sets×30 s
Vertical jumps	2 sets×30 s
Part 3: Running exercises	2 min
Across pitch	2 sets×30 m (75%–80% max)
Bounding	2 sets×30 m
Plant and cut	2 sets (80%–90% max)

WU+S performed all parts, WU-S performed only parts 1 and 3.

### Testing procedures/outcomes

All participants had one familiarisation session before the first test day, where they performed all the tests and five repetitions maximum of the Nordic hamstring exercise. Test days started with the isokinetic dynamometer and countermovement jump (CMJ) tests before performing the 11+ with or without the strength training part. After the warm-up, RPE and training readiness were assessed, followed by sprint, isokinetic dynamometer and CMJ tests.

Maximal concentric knee extensor and flexor torque were tested unilaterally in an isokinetic dynamometer (Humac NORM, CSMi, Stoughton, Massachusetts, USA). Players were seated with the backrest at 85° and the dynamometer aligned with the knee joint axis. Straps were placed across the chest, waist and thigh to isolate the knee extension-flexion movement. The first test was concentric knee extension and flexion at 60°/s. Range of motion was 90°–0° knee flexion. Four warm-up repetitions with increasing intensity preceded four repetitions with maximal effort. The same sequence was performed at 180°/s, and the series was separated with 30 s of rest. We extracted pre-warm-up and post-warm-up peak torque, work per repetition and angle of peak torque for both legs and analysed the averaged values of the legs.

CMJ was measured on a portable force platform (HUR Labs, FP4, Tampere, Finland; maximal sampling frequency 1200 Hz). Players performed three jumps separated by a 30 s break, with hands on their hips and self-preferred squat depth. We extracted pre-warm-up and post-warm-up jump height, peak power, average peak power, peak force and average peak force from the highest jump for analysis.

The 40 m sprint was tested on an indoor running track. Wall-mounted photocells (Athletics Training System, IC Control Media & Sport, Bromma, Sweden) placed 1 m above the ground every 10 m. Players started each sprint standing with the front foot placed 30 cm behind the first photocell. The three trials were separated with a 1 min active break walking back. We retained sprint times for every 10 m (s) from the fastest 40 m for analysis.

A ‘readiness to train’ and ‘RPE’ questionnaire was verbally presented to players immediately after completing the full warm-up. The questionnaire consisted of two Likert-scale questions. Players were asked to answer a number ranging from 1 to 10 on the following two questions: (1) ‘On a scale from 1 to 10, how physically ready do you feel to perform your best if you were to complete a football training session right now?’ and (2) ‘On a scale from 1 to 10, how physically demanding did you feel the warm-up was?’.

### Statistical analysis

Values are expressed as mean±SD and 95% CIs from pretest to post-test. All statistical analyses were completed using SPSS (SPSS V.24, IBM). For the isokinetic dynamometer and CMJ tests, we calculated the absolute change in the outcome variables from pretest to post-test for both warm-up protocols. We assessed the differences using a paired sample t-test. The difference in sprint performance, RPE and training readiness after the two warm-up protocols were analysed with a paired sample t-test. An α level of 0.05 was considered significant.

## Results

Of the 20 players included in the study, 5 players dropped out due to injury (n=2), sickness (n=2) or not showing up to testing (n=1), leaving 15 players (15.7±1.6 years, 167.4±3.6 cm, 59.8±6.2 kg) qualified for analysis.

### Isokinetic strength

One was significantly different out of the six variables tested on the knee flexors. There was a significant difference in the change in hamstring peak torque at 180°/s between the two conditions (p<0.05), with changes favouring the WU-S (table 2).

**Table 2:** Mean difference in pretest and post-test for peak torque, angel of peak torque, work and CMJ measurements

	WU+S			WU-S			
	Pretest	Post-test	Mean diff (95% CI)	Pretest	Post-test	Mean diff (95% CI)	P value
**PT** 60°/s Q	134±26	134±31	0 (−6 to 6)	137±28	137±28	−1 (−5 to 4)	0.82
60°/s H	100±16	95±17	−5 (−8 to −2)	99±17	96±18	−3 (−5 to 0)	0.17
180°/s Q	91±19	89±17	−2 (−5 to 0)	91±17	91±17	0 (−3 to 4)	0.23
180°/s H	76±13	73±12	−3 (−4 to −1)	75±13	74±14	−1 (−2 to 2)	0.034*
**PTA** 60°/s Q	50±7	51±7	2 (−1 to 4)	48±6	51±6	3 (1 to 4)	0.41
60°/s H	24±3	23±3	−1 (−2 to 1)	25±4	25±4	0 (−2 to 2)	0.51
180°/s Q	48±5	50±7	2 (−1 to 6)	47±6	48±5	1 (−1 to 3)	0.39
180°/s H	36±4	36±4	0 (−2 to 2)	36±4	35±4	−1 (−3 to 1)	0.35
**WORK** 60°/s Q	149±29	144±31	−6 (−10 to −2)	150±27	150±27	−1 (−5 to 4)	0.13
60°/s H	112±19	104±19	−8 (−11 to −4)	110±18	105±19	−5 (−9 to – 2)	0.35
180°/s Q	97±20	91±19	−6 (−9 to −3)	96±17	96±17	0 (−4 to 4)	0.019*
180°/s H	81±14	77±13	−3 (−5 to −1)	80±13	79±15	−1 (−3 to 1)	0.10
**CMJ (N=14†)** Height (cm)	26.8±4.1	27.0±4.3	0.3 (0.3 to 0.8)	26.9±4.2	26.9±4.0	0.1 (−0.6 to 0.8)	0.68
PP	2288±419	2274±437	−14 (−70 to 42)	2291±467	2301±416	15 (−54 to 84)	0.55
Average PP	2258±411	2256±432	−2 (−42 to 39)	2270±474	2274±418	4 (−49 to 56)	0.89
PF	1247±202	1209±199	−38 (−80 to 4)	1288±218	1253±217	−35 (−73 to 4)	0.92
Average PF	1238±197	1210±192	−29 (−67 to 10)	1272 ± 226	1251 ± 217	−21 (−46 to 4)	0.76

*Significant differences from pre-test to post-test between warm-up protocol (p<0.05).

†(N=14) = 1 player lost due to measurement error.

CMJ, countermovement jump; Mean diff, mean difference; H, hamstrings; average PF, peak force average; PF, peak force; average PP, peak power average; PP, peak power; PT, peak torque; PTA, angle of peak torque; Q, quadriceps; WORK, work per repetition.

Changes in quadriceps work per repetition at 180°/s also favoured WU-S, with a significant difference between the two groups (p<0.05; table 2). No difference was observed in the other five variables.

### Countermovement jump

No significant differences between warm-up protocols were observed in any outcome measurements in the CMJ test (table 2).

### Sprint performance

**Figure 1:**
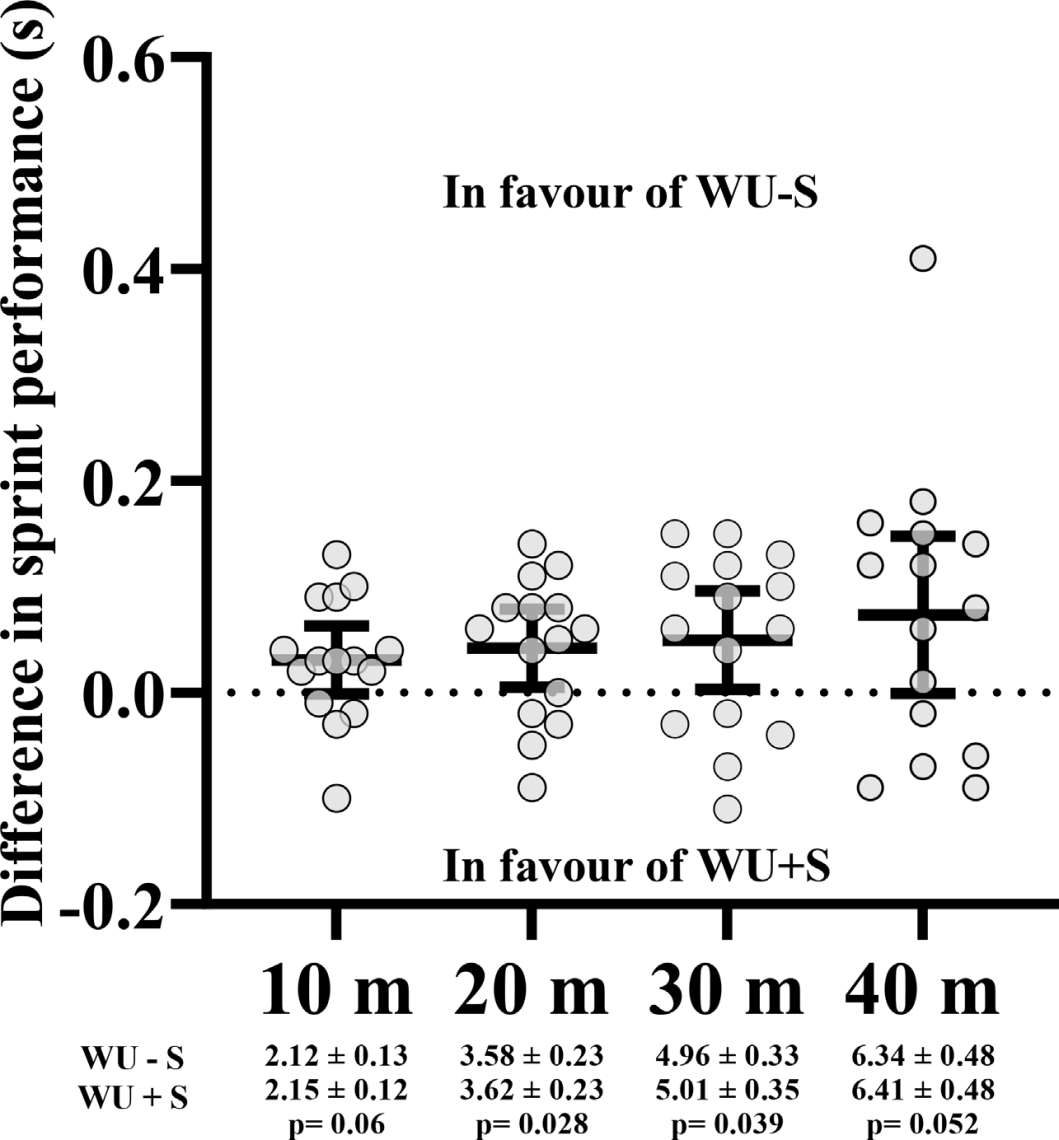
Absolute differences in sprint times comparing WU+S to WU-S. A positive value means WU-S was better than WU+S. Results are presented as mean, 95% CI and SD. Significant difference between warm-up protocols (p<0.05).

Players sprinted significantly faster at 20 m (WU-S: 3.58±0.23, WU+S: 3.62±0.23, p=0.028) and 30 m (WU-S: 4.96±0.33, WU+S: 5.01±0.35, p=0.039) after the WU-S compared with WU+S (figure 1).

### RPE and readiness questionnaire

Players rated their perceived exertion higher in the WU+S than WU-S (WU+S: 6.73±1.16; WU-S: 5.27±1.39, p<0.001). No difference was recorded in the player’s readiness to train after the warm-ups (WU+S: 7.60±1.30; WU-S: 7.93±0.96, p>0.05).

## Discussion

This study evaluated the acute performance effects of the 11+ warm-up protocol with and without the strength training part. Our main finding was that the 11+ strength training part reduced isokinetic hamstring strength at 180°/s and reduced 20 and 30 m sprint performance among female junior football players.

Although not every metric was impaired, the reduction in sprint performance and explosive strength found is significant as both factors are vital for football performance. These reductions demonstrate that the strength training part may be inappropriate to perform before the training session, as optimising football performance needs to be the primary goal.^22^

Our results align with those of Ayala *et al*, who reported that the 11+ resulted in decreased sprint performance compared with a dynamic warm-up consisting of 16 exercises, including aerobic activities, dynamic stretching and football-specific movements.^17^ The decreased sprint performance and peak torque in the knee flexors we observed are likely due to the eccentric Nordic hamstring exercise with maximal effort. One possible mechanism is selective fatigue or damage to the type II muscle fibres.^23 24^ Type II fibres are critical for sprint performance and especially prone to structural damage during heavy eccentric exercise.^25–28^

Conversely to our findings, Bizzini *et al*^18^ and Robles Palazón *et al*^19^ observed sprint changes favouring the 11+ (with the strength training part) compared with a control period and a ‘regular warm-up’, respectively. This may be related to the execution of the strength training part (effort) and how accustomed the players were to the exercises, as female athletes in this age group might have less experience with strength training.^29^ Moreover, this study is the first to test female players.

We observed no difference in the CMJ test, which aligns with a previous study comparing the 11+ with a dynamic warm-up.^30^ Our findings do not align with Bizzini *et al*^18^ reporting that the 11+ significantly reduces CMJ jump height.^18^ One reason we observed a decline in sprint results, but not the CMJ, is that the hamstring muscles are much more important for sprinting than vertical jumping.^31^ The programme has no exercises expected to require near maximal effort for either the quadriceps or the gluteal muscles. It does not fatigue these muscle groups as much as the hamstrings.^32 33^ The Nordic is a specific hamstring exercise, and as expected, peak torque at 180°/s was reduced more after WU+S than WU-S. Peak torque at 60°/s was reduced after WU+S but not different from WU-S. This discrepancy could be due to low statistical power but may also be explained by the eccentric contractions during the Nordic exercise. Eccentric exercise has previously been shown to affect type II muscle fibres more than type I fibres, and thus, the force decrements are more pronounced at higher shortening velocities.^34^

Our RPE results align with those of Chen *et al*, displaying a significantly higher RPE score after completing the 11+ compared with to a dynamic warm-up routine consisting of jogging and dynamic lower extremity exercises.^20^ Although the strength training part increased RPE, there was no difference in readiness to perform. This discrepancy may be due to player mentality and players thinking they should be ready to play following a warm-up as is expected. Furthermore, the Nordic exercise is purely eccentric and has a low metabolic cost.^35^ This may have contributed to the feeling of readiness shortly after the exercise, even if the muscle function was impaired.

Previous intervention studies reported similar strength gains over 12 weeks when the strength training part of the 11+ and the Nordic hamstring exercises were conducted before or after football practice.^16 36^ Furthermore, it has been reported after an 8-week intervention period that eccentric and concentric hamstring strength is better preserved (less fatigue) during a simulated football match if the Nordic exercise is performed after practice.^37^ Performing the exercise in a state of football-specific fatigue may have implications for injury prevention and aid performance in maintaining players’ ability to run, sprint, jump and tackle during later stages of match-play when fatigue occurs. Therefore, strength training may be better suited after rather than before practice.

## Limitations

A limiting factor was that the players were not accustomed to performing the Nordic exercise. Therefore, we do not know the implications if the exercise is performed regularly. On the other hand, to ensure DOMS and the repeated bout-effect did not affect the results. The participants performed both the tests and strength exercises as a familiarisation 1 week before the study started.

Postactivation potential acutely enhances muscular performance and is reported to dissipate after 1–8 min.^38 39^ We might have missed the potentiating effect of either warm-up on the isokinetic and CMJ test. However, potentiated effects after warm-up routines have mainly been found after ballistic and weight-based exercises,^40–42^ neither included in the 11+programme. Therefore, we find it unlikely to have missed any potentiating effect.

Physical performance can be affected by the difference in circadian rhythms, known as the time-of-day effect.^43 44^ No players were tested in the morning (between 06:00 and 10:00 hours) when anaerobic performance was reported to be most affected.^44^ We consider this unlikely to have affected testing.

The difference in the age range in this sample size may have meaning for the results, as significant adaptions in muscle strength occur during adolescence.^45^ Furthermore, our results may be explained by many of the players who are still developing physically. Players might not have been able to exploit their true force potential, affecting the ability to differentiate between each test.

Five players dropped out, which reduces statistical power. However, the analysed sample size was similar to those previously used in warm-up studies.^17 18 46 47^ The difference in dosage between the warm-ups, given the decreased duration when part 2 is removed, should be noted as it may have impacted the outcomes. Moreover, we acknowledge a selection bias in our population, as players were included based on the author’s network. Furthermore, due to a scheduling conflict, we used the project’s two different HUMAC dynamometers (but of the same model). However, the strength results are consistent with minimal differences observed in the pre–post test results, indicating that the results are valid.

## Conclusion

The findings of this study were that performing the 11+ warm-up with the strength training part can impair subsequent knee flexion torque at high velocity and sprint performance in female junior football players.

## Practical applications

The performance decrements in sprint performance and increased perceived rating of exertion by players after warm-up, with strength exercises found in this study, should interest practitioners and researchers. Considering the importance of sprint performance in football as it precedes almost every game-winning action, the results of this study supply valuable information for coaches in the planning of sessions and periodisation of the football season. Implementation of injury preventive exercises, such as those in the strength training part of the 11+ programme, should still be performed. However, there is evidence that rescheduling the strength training part to the end of training increases dose exposure and maintains the efficacy of the 11+.^48^ This study adds reason to believe that the strength training part would be better suited at the end of training as the programme’s injury preventive effect would remain without jeopardising performance.

## Data Availability

Data are available on reasonable request.
